# Effects of Incorporating High-Volume Fly Ash into Tricalcium Silicate on the Degree of Silicate Polymerization and Aluminum Substitution for Silicon in Calcium Silicate Hydrate

**DOI:** 10.3390/ma10020131

**Published:** 2017-02-04

**Authors:** Sungchul Bae, Rae Taylor, David Kilcoyne, Juhyuk Moon, Paulo J. M. Monteiro

**Affiliations:** 1 Department of Architectural Engineering, Hanyang University, Seoul 04763, Korea; 2 Department of Civil and Environmental Engineering, University of California, Berkeley, CA 94720, USA; raemorristaylor@gmail.com (R.T.); monteiro@berkeley.edu (P.J.M.M.); 3 Lawrence Berkeley National Laboratory, 1 Cyclotron Road, Berkeley, CA 94720, USA; alkilcoyne@lbl.gov; 4 Department of Civil & Environmental Engineering, National University of Singapore, 1 Engineering Drive 2, Singapore 117576, Singapore; ceemjh@nus.edu.sg

**Keywords:** calcium silicate hydrate, tricalcium silicate, fly ash, hydration products, X-ray microscopy

## Abstract

This study assesses the quantitative effects of incorporating high-volume fly ash (HVFA) into tricalcium silicate (C_3_S) paste on the hydration, degree of silicate polymerization, and Al substitution for Si in calcium silicate hydrate (C–S–H). Thermogravimetric analysis and isothermal conduction calorimetry showed that, although the induction period of C_3_S hydration was significantly extended, the degree of hydration of C_3_S after the deceleration period increased due to HVFA incorporation. Synchrotron-sourced soft X-ray spectromicroscopy further showed that most of the C_3_S in the C_3_S-HVFA paste was fully hydrated after 28 days of hydration, while that in the pure C_3_S paste was not. The chemical shifts of the Si K edge peaks in the near-edge X-ray fine structure of C–S–H in the C_3_S-HVFA paste directly indicate that Al substitutes for Si in C–S–H and that the additional silicate provided by the HVFA induces an enhanced degree of silicate polymerization. This new spectromicroscopic approach, supplemented with ^27^Al and ^29^Si magic-angle spinning nuclear magnetic resonance spectroscopy and transmission electron microscopy, turned out to be a powerful characterization tool for studying a local atomic binding structure of C–S–H in C_3_S-HVFA system and presented results consistent with previous literature.

## 1. Introduction

High-volume fly ash (HVFA) concrete, in which more than 50 wt % of Portland cement (PC) is replaced by fly ash, has been successfully employed in building structures to mitigate CO_2_ emissions during PC clinker production [1]. A significant problem with HVFA concrete is that its initial strength development rate is slower than that of PC concrete due to its lower cement content [2]. However, HVFA enhances properties of concrete, such as its workability, durability, and long-term compressive strength, and performance losses during the early ages can be overcome by optimizing the particle size distribution of the fly ash and the concrete mix proportions [3,4,5]. The enhanced properties of concrete due to HVFA incorporation result from an increase in the number of nucleation sites for PC hydrates [4,5] and from the pozzolanic reaction between Ca(OH)_2_ and the amorphous aluminosilicate-rich ash [6,7].

Calcium silicate hydrates (C–S–H), the primary binding phase in the PC system, influence both the mechanical and chemical properties of the PC paste. The C–S–H characteristics are affected by several factors, such as the PC chemical composition, the water-to-cement (W/C) ratio and the introduction of supplementary cementitious materials (SCMs), including fly ash [8,9,10]. In C–S–H formed in pure PC systems, it is known that the tetrahedral SiO_4_^4−^ units are balanced by the presence of calcium cations and that defective sheets of the silicate chains are formed by silicate polymerization during the PC hydration process [11]. C–S–H in the pure PC systems is generally poorly crystalline, and its Ca-to-Si molar ratio (Ca/Si ratio) ranges from 1.2 to 2.1 with a typical value of approximately 1.75 [12]. In PC-SCM systems, silica-rich SCM leads to a lower Ca/Si ratio in C–S–H than that in the pure PC system [9,10,13], and its crystal structure is similar to that of 14 Å tobermorite [14]. This structure is referred to as C–S–H(I) and consists of longer, more ordered silicate chains compared with the C–S–H structure produced in the pure PC system [10,15]. These characteristics are due to the enhanced degree of silicate polymerization caused by the abundant silicate from the SCM sources. When C–S–H has a low Ca/Si ratio, aluminum (Al) substitution for silicon (Si) at the bridging tetrahedral sites in the C–S–H silicate chain increases [16], resulting in Al-substituted C–S–H (C–A–S–H) [17]. Al incorporation in C–S–H depends on the total amount of Al present in the system. A previous study showed that synthetic C–S–H with Ca/Si of 1.0 incorporates Al resulting in C–A–S–H formation in which Al/Si is less than 0.1 [18]. However, at higher Al/Si (≥0.1), strätlingite or katoite could be precipitated in addition to C–A–S–H [13]. Thermodynamic modeling [10] also suggests that calcium aluminate silicate hydrate phases might precipitate and intermix with C–A–S–H or C–S–H(I) when the amount of alumina exceeds that accommodated by C–S–H in the HVFA system. A synchrotron micro X-ray diffraction study [14] exhibited the co-existence of strätlingite and C–S–H(I) in a PC-HVFA system and proved that the total amount of the alumina and silicate phases provided by the HVFA is a major factor in the formation of the phases. Al uptake in C–S–H also leads to structural changes in synthetic C–S–H, resulting in an increase in the chain length and the basal d-spacing (002) [18].

Both the degree of silicate polymerization and the Al substitution for Si in C–S–H are the main factors that affect the Ca/Si ratio and the Si binding energy in C–S–H. Chemical shifts in the Ca and Si binding energies caused by the higher degree of silicate polymerization have been studied by X-ray photoelectron spectroscopy (XPS) [19,20] and X-ray absorption spectroscopy (XAS) [21]. Reinheiemer and Casanova [19] observed that the Ca/Si ratio in C–S–H of C_3_S thin films decreases as the C–S–H silicate chain length and SiO_2_ content increase during the silicate polymerization. Li et al. [21] demonstrated that the Si binding energy decreases when Al substitutes for Si in aluminosilicate minerals. Experimental results [12,18,22,23,24,25,26] and a theoretical model based on *ab* *initio* calculations [27] demonstrated that Al ions prefer bridging chain sites to non-bridging sites in C–S–H because the bridging tetrahedral sites are charge balanced by monovalent alkali and interlayer ions such as Na^+^, H^+^, and Ca^2+^.

Likewise, in the HVFA system, the Si binding energy in C–S–H is expected to increase due to the silicate polymerization process induced by the presence of additional silicate provided by HVFA. Alternatively, the Si binding energy in C–S–H is expected to decrease due to Al substitution for Si in C–S–H [21]. The detailed investigations of the hydration kinetics, C–S–H morphology and atomic binding structure in the C_3_S-HVFA system are crucial for understanding and optimizing the HVFA system eventually improving the performance of the HVFA concrete. The effects of SCMs on the silicate chain length and Al substitution for Si in C–S–H have been quantitatively studied by powder characterization methods such as nuclear magnetic resonance (NMR) spectroscopy [23,24,28]. However, the characterization techniques using powdered samples are for bulk analysis and provide limited local information at the micro- or nanoscale. Scanning transmission X-ray microscopy (STXM), is a soft X-ray spectromicroscopy technique that can be used for morphological imaging at a spatial resolution of 30 nm and for near-edge X-ray fine structure (NEXAFS) analysis of a specific area in the X-ray microscopy image with a high spectral resolution of 0.1 eV. Recently, this technique was used to successfully investigate the effects of the degree of silicate polymerization on the chemical shifts of C_3_S hydration products [29,30] and of C–S–H synthesized with different Ca/Si ratios at the Ca L_III,II_ edge and Si K edge in the NEXAFS spectrum [29]. This powerful technique also enables to characterize the kinetics of early age cement clinker hydration in situ by employing a wet cell [31]. Interpretations derived from STXM analysis, therefore, could be supplementary information regarding the effect of HVFA on both morphology and atomic binding structure of C–S–H.

The purpose of the present paper is to demonstrate the effects of HVFA on C_3_S hydration by examining the hydration kinetics, morphology of the hydration products, and atomic binding structure of C–S–H. Pure triclinic C_3_S, which has been suggested to be the primary clinker phase in PC, was utilized with and without 50 wt % Class F fly ash. STXM was employed to examine the morphological and atomic binding structural differences in a local area of C–S–H in the pure C_3_S and C_3_S-HVFA systems. The NEXAFS of the Ca L_III,II_ edge, Al, and Si K edge was analyzed to quantitatively determine the effect of HVFA on silicate polymerization and Al incorporation into C–S–H. The hydration products in the HVFA system were identified, and the amount of Ca(OH)_2_ was determined by synchrotron powder X-ray diffraction (XRD) and thermogravimetric analysis (TG/DTG). In addition to XRD and TG/DTG, isothermal conduction calorimetry was performed to monitor the rate of heat evolution in the early hydration process. ^27^Al and ^29^Si magic-angle spinning nuclear magnetic resonance (MAS NMR) spectroscopy was employed to confirm Al incorporation into C–S–H and determine the degree of silicate polymerization by comparing the C–S–H silicate chain length in the pure C_3_S and C_3_S-HVFA systems. The morphological details and Ca/Si ratio of C–S–H in the C_3_S-HVFA paste were investigated by transmission electron microscopy (TEM) with energy-dispersive X-ray spectroscopy (EDX). Based on the experimental results, the schematic structure of C–S–H in the HVFA system was suggested.

## 2. Experimental Procedure

### 2.1. Materials and Sample Preparation

Triclinic C_3_S and Class F fly ash which contains a high amount of SiO_2_ (62.00 wt %) and Al_2_O_3_ (18.90 wt %) but less CaO (5.98 wt %), were used as raw materials (see Table 1). C (pure C_3_S) and C-FA (50 wt % C_3_S, 50 wt % fly ash) pastes were prepared by hand-mixing the raw materials with distilled water. For all samples, the water-to-solid ratio was maintained at 0.5. The hand-mixed pastes were cast into PELCO^®^ disc block embedding molds (ϕ18 × 3 mm) and cured at 23 ± 1 °C and 95% ± 5% relative humidity. The pastes were demolded after 1, 3, 7, or 28 days, and ethanol was used to stop the hydration process. The samples were then kept under vacuum in carbonation-free conditions until testing. For isothermal conduction calorimetry, two test samples containing 1 gram C_3_S and 0 or 50% fly ash were prepared with a water-to-solid ratio of 0.5. Finely ground powder samples were used for the XRD, TG/DTG and TEM experiments. All samples were stored in a vacuum bag and ground just before measurement to minimize their carbonation.

For the STXM measurements, thin-sectioned samples (~200 μm thick slices) were cut from the hardened pastes with a diamond wafering blade and then mechanically ground and polished. Kerosene was used to avoid any further hydration of the samples during these procedures. A small piece (~3 × 3 mm^2^) of the thin-sectioned sample was placed on a copper grid with a single slot in the center for ion milling. The sample was ion milled by PIPS™ at the National Center for Electron Microscopy (NCEM), and stored in a box, and sealed in a vacuum bag. The details of the sample preparation for the TEM and NMR experiments are described in later sections.

### 2.2. Instrumental Methods

#### 2.2.1. Monochromatic Synchrotron X-ray Diffraction (XRD)

XRD experiments were performed at beamline 12.2.2 of the ALS at LBNL. This beamline uses a monochromatic synchrotron X-ray (λ = 0.6199 Å) focused to an area of 10 × 10 µm^2^. To calibrate the working distance between the sample and the diffraction detector, a LaB_6_ powder diffraction standard was used. The collected two-dimensional XRD patterns using an MAR345 image plate (3450 × 3450 pixels) were converted into one-dimensional intensity versus 2-theta scans using the Fit2D program [32].

#### 2.2.2. Scanning Transmission X-ray Microscopy (STXM)

STXM data were collected at ALS beamlines 5.3.2.1 and 5.3.2.2 with the synchrotron storage ring operating at 1.9 GeV and 500 mA. The counting times were on the order of a few milliseconds or less per pixel and the total counting times for each single image occurred at a frequency of every few minutes depending on the number of total pixels. Four types of images were obtained: (1) single images; (2) image contrast maps; (3) image stacks; and (4) RGB overlay maps*.* The details of STXM images can be found elsewhere [29,30,31]. The RGB overlay maps were created by singular value decomposition (SVD) based on reference spectra [29,33]. CaCO_3_, CO_2_, quartz, and 100 nm-thick aluminum foil were used as the references for the Ca L, Si K, and Al K edges, respectively, to calibrate the energy positions to maintain consistency with previous studies [29,31,34,35].

#### 2.2.3. ^27^Al and ^29^Si Magic-Angle Spinning Nuclear Magnetic Resonance (MAS NMR) Spectroscopy

For NMR measurements, 28 day-hydrated C and C-FA were finely ground and evenly packed into a 3.2 mm zirconia rotor, and the open end was sealed with a Vespel cap. The rotor was spun at 20 kHz using a Bruker UltraShield 600 WB Plus with a 14.1 T magnet operating at 156.40 MHz for ^27^Al and at 119.23 MHz for ^29^Si. For both sets of experiments, the dwell time was 7.0 µs, and 1024 and 4096 acquisitions were collected in the ^27^Al and ^29^Si measurements, respectively. The magic angle was set to 54.734° in both cases and KBr was used as the reference. For ^27^Al MAS NMR, the samples were spun for no longer than 40 min to prevent dehydration and any consequential loss in peak intensity [36].

^27^Al MAS NMR spectrum can be difficult to interpret because ^27^Al is affected by quadrupolar interactions, leading to peak broadening as well as a field-dependent shift in the resonance position [23,37]. Whereas high-quality ^29^Si NMR spectrum require long acquisition times, the acquisition time for ^27^Al is much shorter less than the former due to its spin nuclei of I = ^5^/_2_ and natural abundance of 99.99%. Thus, good-quality spectrum can be obtained even from time-limited measurements. In previous works that utilized the same equipment, the probe exhibited a large Al background [38]. For this reason, a sample containing only C_3_S, which should have no Al signal, was analyzed using the same acquisition setup as the other samples based on the assumption that the signal obtained would be the relevant background signal and could be removed from the other spectrum. However, no signal was detected, and therefore, no background removal was necessary.

The mean chain length (MCL) of C–S–H formed in the C and C-FA paste along with the Al/Si ratio of C–S–H in the C-FA paste were calculated using the following equations based on the assumption that Al did not occupy end-chain tetrahedral sites [12].

(1) $${\bar{MCL}}_{\left(C\right)}=\frac{2}{\frac{Q^{1}}{\left(Q^{1}+Q^{2}\left(0Al\right)\right)}}$$

(2) $${\bar{MCL}}_{\left(C-FA\right)}=\frac{2}{\frac{Q^{1}}{\left(Q^{1}+Q^{2}\left(0Al\right)+\frac{3}{2}Q^{2}\left(1Al\right)\right)}}$$

(3) $$Al/Si_{\left(C-FA\right)}=\frac{\frac{1}{2}Q^{2}\left(1Al\right)}{\left(Q^{1}+Q^{2}\left(0Al\right)+Q^{2}\left(1Al\right)\right)}$$

#### 2.2.4. Transmission Electron Microscopy (TEM) Imaging

TEM imaging was performed using a Titan G2 80-300 kV microscope (FEI Company, Hillsboro, OR, USA) equipped with a 4 k × 4 k CCD camera (Gatan, Pleasanton, CA, USA) and EDX detector operating at 300 kV. The imaging/X-ray analysis was performed using the ISIS software (Oxford Instruments, Oxford, UK). The powder of the C-FA paste hydrated for 28 days was suspended in ethanol, and 2 µL of the suspension was deposited on a holey carbon film and dried for five minutes before TEM observation. The selected area electron diffraction (SAED) technique was employed to determine the crystallinity of each area before chemical analysis. 

#### 2.2.5. Thermogravimetric Analysis (TG/DTG) and Isothermal Conduction Calorimetry

TG/DTG tests were performed using ground C and C-FA samples that were aged for 1, 3 and 28 days. The samples were heated from room temperature to 1000 °C at a rate of 10 °C/min. Approximately 25 mg of finely ground powder was placed in a ceramic crucible and heated in Exstar 6300 TG/DTA instrument (Seiko Instruments Inc., Chiba, Japan). The nitrogen gas flow rate was 50 mL/min.

The heat flow of C and C-FA in the early age was monitored in an isothermal conduction calorimeter (TAM-air, TA instruments, New Castle, DE, USA) at a constant temperature of 23 °C. Deionized water and powder was hand-mixed inside of a glass ampule for 2 min. The paste was tightly sealed immediately after mixing and placed in the isothermal calorimeter. For the powder containing 50% fly ash, C_3_S and fly ash were hand-mixed for a minute before adding water.

## 3. Results and Discussion

### 3.1. XRD Results

**Figure 1:**
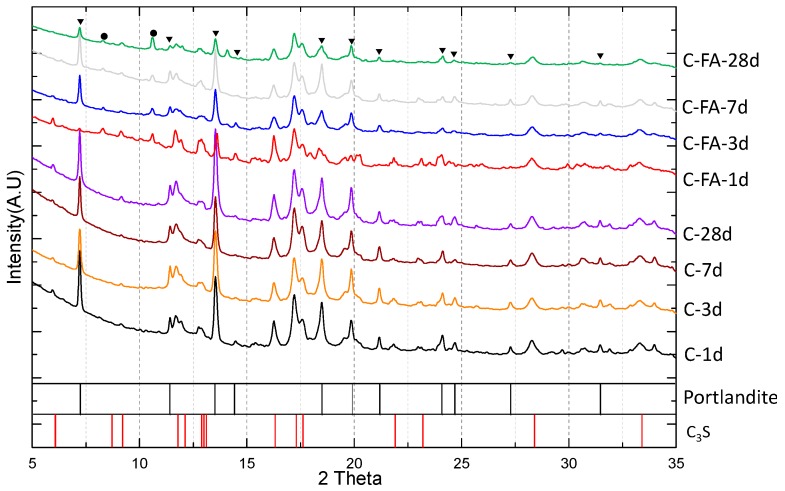
Synchrotron powder X-ray diffraction patterns for C_3_S (C) and C_3_S substituted 50% by fly ash (C-FA) pastes. ●: Quartz, ▼: Portlandite (Ca(OH)_2_). Relative intensities above 5% were classified using the ICDD PDF2 database reference 00-087-0674.

Figure 1 presents the XRD patterns of the pure C_3_S and C_3_S-HVFA pastes after 1, 3, 7 and 28 days of curing. The XRD patterns contained few unreactive crystalline quartz phases, anhydrous C_3_S or Ca(OH)_2_. No other phases, such as C–S–H(I), AFt, AFm, monocarbonate, or strätlingite, were observed. The Ca(OH)_2_ peak intensity of the C_3_S-HVFA paste was less than that of the pure C_3_S paste due to the dilution of C_3_S by fly ash. The Ca(OH)_2_ peak intensity of the pure C_3_S system increased as the hydration period increased from 1 day to 28 days. However, in the C_3_S-HVFA system, the Ca(OH)_2_ peak intensity decreased as the hydration period increased from 7 days to 28 days because of the pozzolanic reaction between Ca(OH)_2_ and the aluminosilicate phase.

### 3.2. TG/DTG and Isothermal Conduction Calorimetry Results

**Figure 2:**
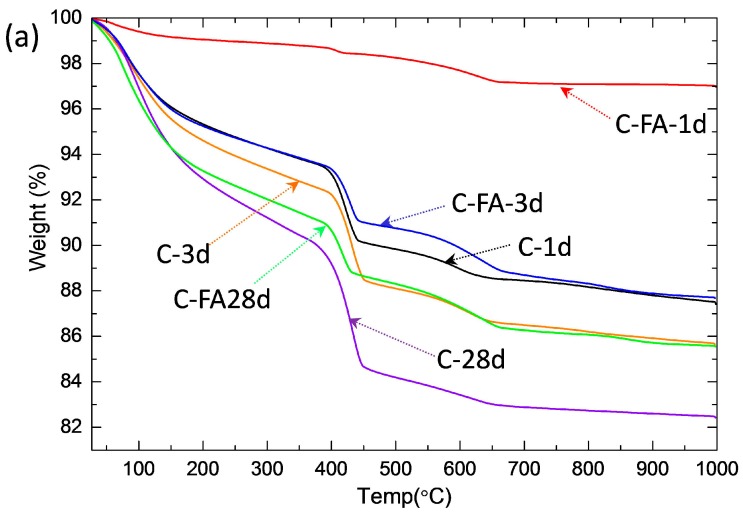

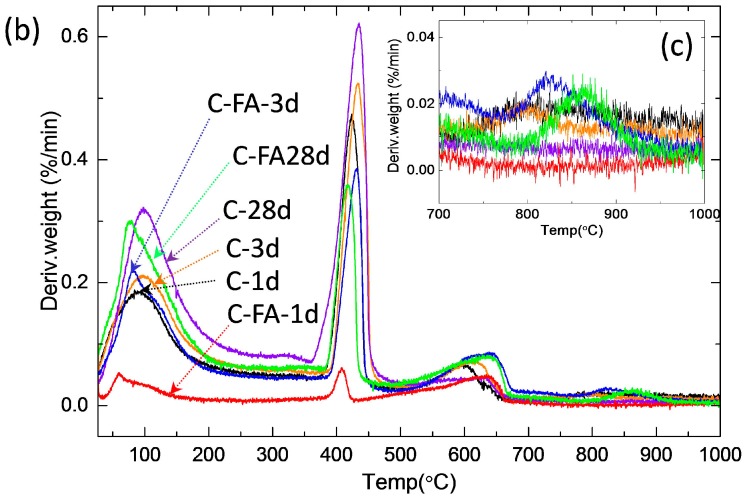
Results of thermal analysis. (**a**) TG and (**b**) DTG results of the C and C-FA.

**Figure 3:**
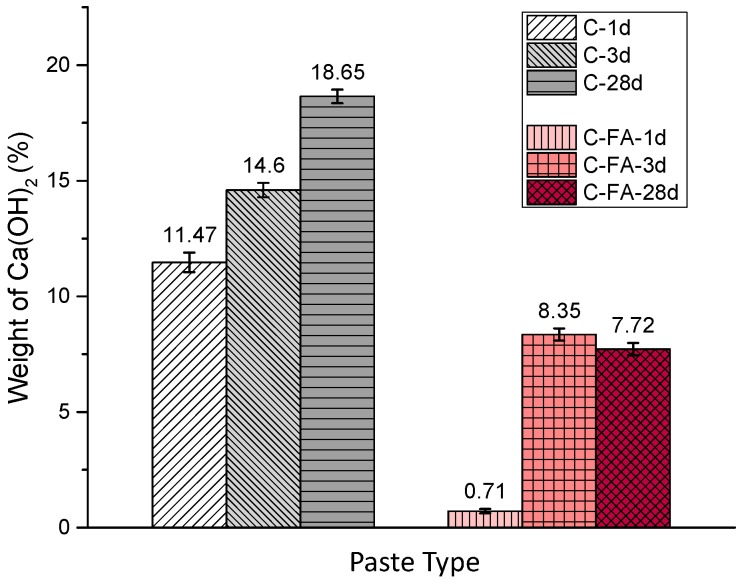
Amount of produced Ca(OH)_2_ as a percentage of total weight in C and C-FA. The Ca(OH)_2_ content was normalized against the C_3_S content of the pastes.

TG/DTG analysis was performed (Figure 2) to compare the quantity of Ca(OH)_2_. Based on the TG/DTG mass loss, the amount of Ca(OH)_2_ produced in the pure C_3_S and C_3_S-HVFA samples was calculated as a percent of the total weight (Figure 3). The amount of Ca(OH)_2_ in the pure C_3_S pastes gradually increased as the hydration period increased up to 28 days. In contrast to the pure C_3_S results, a very small amount of Ca(OH)_2_ (0.71 wt %) was observed in the C-FA-1d sample, and it increased significantly when the hydration period was increased to 3 days.

**Figure 4:**
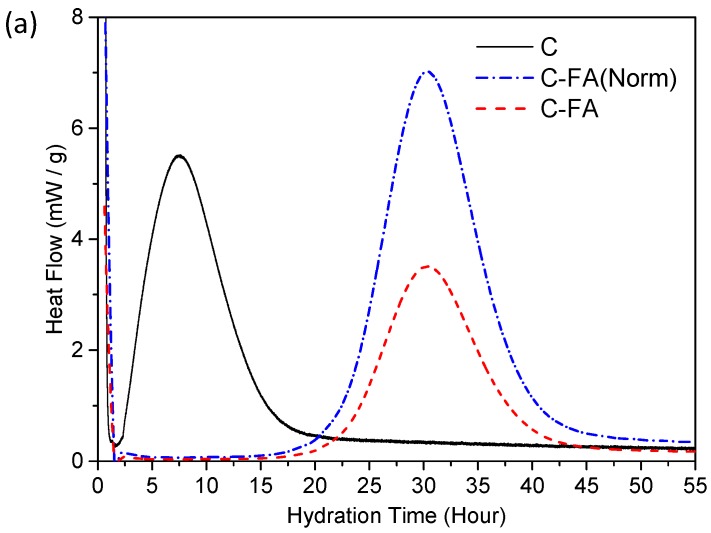

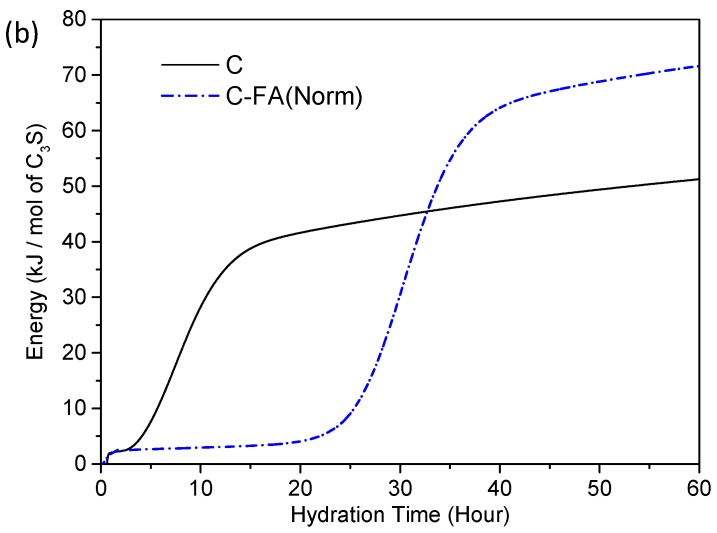
The results of isothermal conduction calorimetry. (**a**) Rate of heat evolution for pure C_3_S(C) and the C_3_S substituted with 50% by fly ash (C-FA). C-FA was normalized against the C_3_S content, as shown in C-FA(Norm); (**b**) Cumulative heat evolved for C and C-FA(Norm).

This retarding effect of fly ash on the early PC hydration has been previously observed [5,39]. To verify the hydration retardation due to HVFA incorporation after 1 day, the rate of heat evolution and the cumulative heat evolved for pure C_3_S and the C_3_S substituted with 50% by fly ash were measured by isothermal conduction calorimetry (Figure 4).

The end of the induction period of C and C-FA occurred at approximately 1.5 and 15 h, respectively. Compared to pure C_3_S, however, the heat flow peak height increased for C-FA per mass of C_3_S after the induction period. As a result, C-FA showed a higher normalized cumulative heat evolution after 33 h relative to pure C_3_S. Based on the results, the degree of C_3_S hydration (α) was obtained by dividing the accumulated heat evolution by the enthalpy of the following C_3_S reaction:

C_3_S(*s*) + (*x*+1.3)H_2_O(*l*)→C_1.7_–S–H*_x_*(*s*) + 1.3Ca(OH)_2_ (*Δ_r_H* = −121 kJ·mol^−1^) (4)

where *x* is the molar water content of C–S–H [40]. 

The α at 24 h for C-FA (α = 0.05) was considerably less than that of C (α = 0.35). However, after 60 h, the α for C-FA (α = 59) significantly increased compared to that of C (α = 43), which is consistent with the results of XRD and TG-DTG at 1 and 3 days. The results of XRD and thermal analysis indicate that the incorporation of HVFA into the C_3_S system significantly retards the early age hydration of C_3_S and extends the induction period of C_3_S resulting in the delay of C–S–H and Ca(OH)_2_ formation before 1 day of hydration. However, HVFA enhances the precipitation and growth of hydration products through the acceleration and deceleration period (the main hydration peak of isothermal calorimetry) as observed in the considerably delayed increase of the Ca(OH)_2_ in C-FA after 3 days. The morphological details of the C_3_S in the pure C_3_S and C_3_S-HVFA systems will be further discussed using STXM analysis.

### 3.3. STXM Analysis

**Figure 5:**
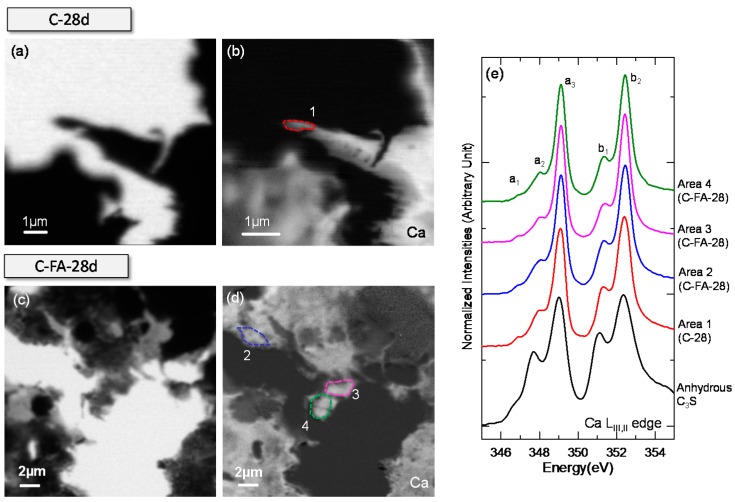
The scanning transmission X-ray microscopy (STXM) results of C-28d. (**a**) Single image of C-28d taken at 349 eV; (**b**) Ca image contrast map of C-28d; (**c**) single image of C-FA-28d taken at 340 eV; (**d**) Ca image contrast map of C-FA-28d; and (**e**) Ca L_III,II_ edge near-edge X-ray fine structure (NEXAFS) spectra of anhydrous C_3_S, C-28d, and C-FA-28d extracted from the ROIs specified in (**b**,**d**).

The STXM images and Ca L_III,II_ edge NEXAFS spectra of C-28d and C-FA-28d are presented in Figure 5. Due to the low X-ray photon energies (340–360 eV) employed in the Ca L_III,II_ edge NEXAFS analysis, the sample areas that were thin enough for X-ray beam penetration and had a uniform Ca distribution in the image contrast map were selected for analysis (Figure 5b,d). The Ca L_III,II_ edge NEXAFS spectra obtained from the regions of interest (ROIs) and those of anhydrous C_3_S [29] are shown in Figure 5e. Each spectrum was normalized to the incident beam intensity (I_0_), and the linear pre-edge background was removed. Two main spin-orbit-related peaks, L_III_ 2p_3/2_ (a_3_) and L_II_ 2p_1/2_ (b_2_), are observed at the Ca L_III,II_ edge of calcium compounds along with several smaller peaks (a_1_, a_2_, b_1_) that precede the main peaks [41]. These multi-peak patterns in Ca L_III,II_ edge NEXAFS are occurred by the crystal field due to the symmetry of the atoms in the first coordination sphere of the Ca cation [42]. The peak positions and energy separations in Ca NEXAFS depend on the Ca symmetry and the magnitude of the crystal field (10Dq) [42]. The peak positions and energy separations of the reference of anhydrous C_3_S reference [29], Ca(OH)_2_ reference [29], and the ROIs in C-28d and C-FA-28d are shown in Table 2. 

As shown in Figure 5e, the Ca L_III,II_ edge absorption features (peak positions and intensity ratios) of anhydrous C_3_S [29] differed from those in the ROIs of C-28d (Area 1) and C-FA-28d (Areas 2-4). The ROIs in C-28d and C-FA-28d exhibited similar absorption features to those in the C–S–H produced by C_3_S hydration and synthesized C–S–H [29]. The decrease in the peak intensity ratio and energy separations (a_3_ − a_2_ and b_2_ − b_1_), which indicate that the crystal field and Ca symmetry were lower, could be caused by C–S–H formation.

In the present study, the peak positions in the Ca L_III,II_ edge of C–S–H in C-28d and C-FA-28d were similar. Previous STXM work [29] demonstrated that synthesized C–S–H with different molar ratios of Ca/Si showed no variation in peak positions or energy separation in the Ca L_III,II_ edge NEXAFS spectra, which implies that the silicate polymerization in C–S–H does not have a great influence on the Ca binding energy in C–S–H. Similarly, in the present study, the additional silicate and Al substitution in the C_3_S-HVFA system did not have a significant effect on the C–S–H Ca L_III,II_ edge NEXAFS spectrum, indicating that the enhanced silicate polymerization and Al uptake in C–S–H have minor effects on the Ca binding energy in the CaO layer of C–S–H.

**Figure 6:**
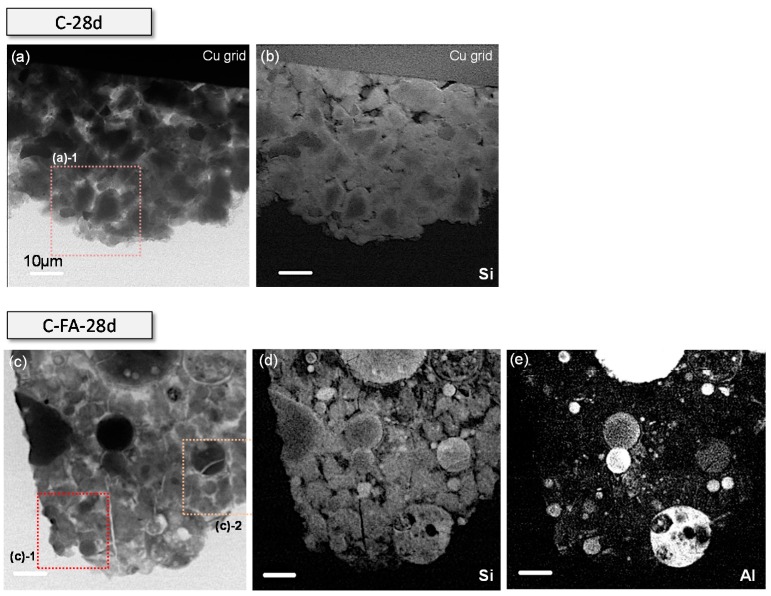
The STXM images of C-28d and C-FA-28d. (**a**) Single image of C-28d; (**b**) Si image contrast map of C-28d; (**c**) single image of C-FA-28d; (**d**) Si image contrast map of C-FA-28d; and (**e**) Al image contrast map of C-FA-28d. Single images were taken at 1840 eV. Image contrast maps were collected by subtracting the single image taken below each edge from above the edges. The brighter areas in the image contrast map (**b**,**d**,**e**) contains higher amounts of each element. The dotted square areas labeled with the index in (**a**,**c**) were magnified and used for NEXAFS analysis.

Si and Al image contrast maps (Figure 6) were generated by subtracting the images taken below the Si and Al K edges, respectively, from those taken above [34,43]. The bright areas in the image contrast maps represent a high concentration of the element [43]. C_3_S particles in C-28d (Figure 6b) had a core area and a Si-rich C–S–H layer that was brighter than the core area. Moreover, the exact boundary between the areas was clearly resolved. It should be noted that the C–S–H layer had a uniform Si concentration, implying that silicate polymerization occurred uniformly throughout the layer, in agreement with a previous STXM study [29].

In the C_3_S-HVFA system, the fly ash particles had greater Si and Al concentrations after hydration for 28 days as shown by the C-FA-28d Si and Al image contrast maps (Figure 6d,e). It should be noted that in contrast to the C_3_S particles in the pure C_3_S system, those in the C_3_S-HVFA system, which did not contain Al as shown by the image contrast map, had a very uniform Si concentration and no obvious C–S–H layer on C_3_S. This was due to the enhanced dissolution of the C_3_S particles in the presence of HVFA, as determined by thermal analysis.

**Figure 7:**
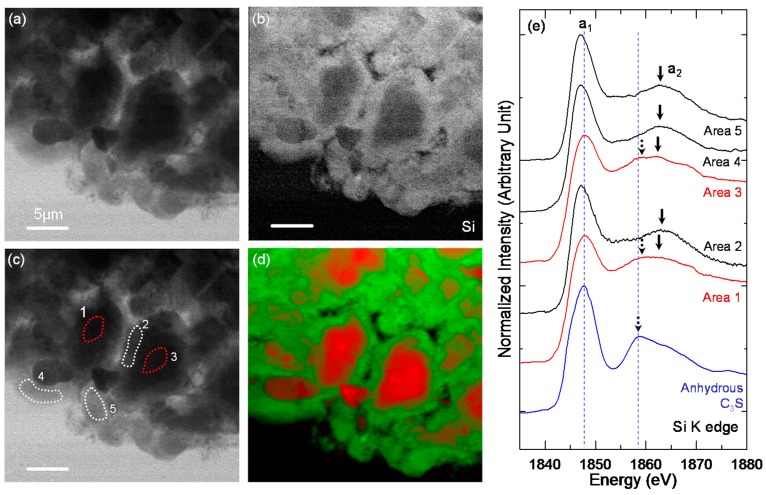
The STXM results of C-28d. (**a**) Single image taken at 1840 eV; (**b**) Si image contrast map; (**c**) areas selected for Si NEXAFS analysis (Area 1 and 3: core area; Area 2, 4 and 5: C–S–H layer); and (**d**) RGB overlay maps assessed by using the spectra obtained from Area 1 (**red**) and Area 2 (**green**), shown in (**e**).

Figure 7 shows the STXM imaging and Si K edge NEXAFS spectroscopy results of the selected areas illustrated by the dotted square in Figure 6a. In the Si K edge NEXAFS spectrum (Figure 7e), peak a_1_, the main peak, was attributed to the Si K edge induced by the electronic transition from the Si 1s orbital to the anti-bonding t_2_ (3p-like state) orbital. The a_2_ peak was assigned to the multiple scattering effect beyond the second coordination sphere [21]. 

In the present study, the main peak (a_1_) for the C–S–H layer (Areas 2, 4 and 5) on C_3_S had a lower energy than that of anhydrous C_3_S (Figure 7e). Alternatively, the core area of the C_3_S particles (Areas 1 and 3) had a similar or even higher peak energy than anhydrous C_3_S. The broad widths of peaks a_1_ and a_2_ of the core area were due to the transmission X-ray signals penetrating through the core area of C_3_S, which contains both the residual anhydrous C_3_S and C–S–H on C_3_S. 

During C_3_S hydration, the Si in the SiO_4_ monomer that dissolved from the outer area of C_3_S during hydration had a lower binding energy than the Si in anhydrous C_3_S due to the smaller silicate environmental network (Q^n^), resulting in a decrease in the Si K edge energy in the C–S–H layer on C_3_S [29]. In contrast, the a_2_ peak of the C–S–H layer (Areas 2, 4, and 5) was shifted to a higher energy (by +(3.6–4.1) eV) than that of anhydrous C_3_S. Thus, the energy separation between the a_1_ and a_2_ peaks increased. This implies that the contribution from multiple scattering increased more than the Si 1s binding energy due to the higher degree of silicate polymerization leading to a more developed silicate network. The RGB overlay map obtained by SVD using the NEXAFS spectra of Areas 1 (core area) and Area 4 (C–S–H layers) illustrates the distribution of the C–S–H layer and core area. The RGB overlay map clearly differentiated between the uniform C–S–H layers and C_3_S core areas in C-28d (Figure 7d). The C–S–H layer that formed on C_3_S in the early age of hydration retarded the hydration of the C_3_S core area in the pure C_3_S system, resulting in an anhydrous C_3_S core area after 28 days of hydration.

**Figure 8:**
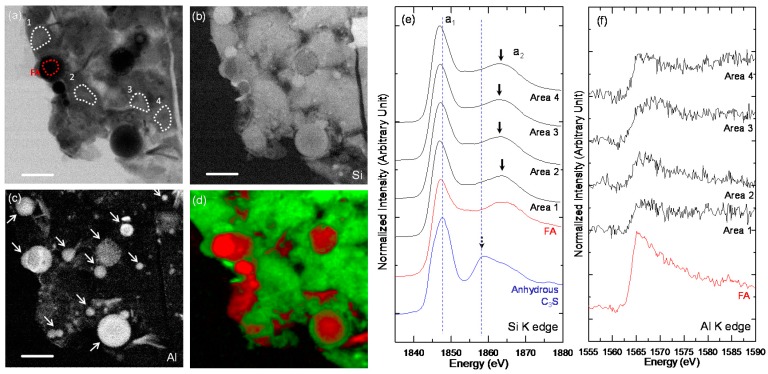
The STXM results of C-FA-28d (Figure 7c-1). (**a**) Single image taken at 1840 eV with marked areas selected for Si NEXAFS analysis (FA: fly ash); (**b**) Si image contrast map; (**c**) Al image contrast map; (**d**) RGB overlay maps obtained using the spectra obtained from FA (**red**) and Area 2 (**green**); (**e**) Si K edge NEXAFS spectra, and (**f**) Al K edge NEXAFS spectra. The Si and Al K edge spectra were obtained from ROIs illustrated in (**a**). Fly ash particles are indicated by the arrows in the Al image contrast map.

**Figure 9:**
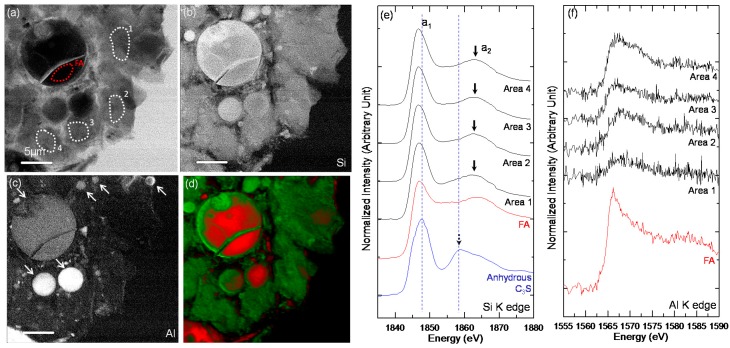
The STXM results of C-FA-28d (Figure 7c-2). (**a**) Single image taken at 1840 eV with marked areas for Si NEXAFS analysis (FA: fly ash); (**b**) Si image contrast map; (**c**) Al image contrast map; (**d**) RGB overlay maps obtained using the spectra from FA (**red**) and Area 2 (**green**); (**e**) Si K edge NEXAFS spectra; and (**f**) Al K edge NEXAFS spectra. The Si and Al K edge spectra were obtained from the ROIs illustrated in (**a**). Fly ash particles are indicated by the arrows in the Al image contrast map.

The single image, Si and Al image contrast maps, and Si and Al K edge NEXAFS analysis of the ROI in C-FA-28d (Figure 6c-1) are shown in Figure 8. The NEXAFS spectra (Figure 8e,f) were obtained from the ROIs illustrated in Figure 7a. As observed in the low-magnification Si image contrast map (Figure 6d), the Si concentration of C–S–H in C-FA-28d, which contained a small amount of Al, appeared to be very uniform and without anhydrous C_3_S. The fly ash particles and the areas assumed to be C–S–H were analyzed by Si and Al NEXAFS spectroscopy to investigate the degree of silicate polymerization in the C_3_S-HVFA system and to compare the spectra to the C–S–H obtained in the pure C_3_S system. The fly ash particles were differentiated from hydrated C_3_S based on their spherical shape and high Al concentration. The a_1_ peak energies of C–S–H were less (from −0.6 to −0.9 eV) than that of anhydrous C_3_S. In addition, the peak energies of the C-FA-28d ROIs were lower (from 0 to −0.3 eV) than those of the C–S–H layer on C_3_S (Area 5 in Figure 7c). Alternatively, the a_2_ peak energies of C–S–H in C-FA-28d were greater (+(0.2–1.2) eV) than those of the C–S–H layer on C_3_S in C-28d. The a_2_ peak of anhydrous C_3_S (1858.9 eV) [29], which was observed in C-28d, was not detected, implying that the C_3_S in C-FA-28d was fully hydrated. In addition, the Al K edge NEXAFS analysis (Figure 8f) shows that C–S–H in C-FA-28d contained much less Al than fly ash particles. The Al traces in C–S–H were assumed to result from the Al incorporation into C–S–H. The RGB overlay map obtained using the NEXAFS spectra of the fly ash and Area 2 is shown in Figure 8d. C–S–H with similar Si K edge NEXAFS features were distributed throughout C-FA-28d, which is consistent with the NEXAFS analysis results. Another area of C-FA-28d (Figure 6c-2) was selected for analysis, and the results are presented in Figure 9. All of the results were highly consistent with those of the selected area shown in Figure 8. No anhydrous C_3_S core area in C_3_S was observed in C-FA-28d.

**Figure 10:**
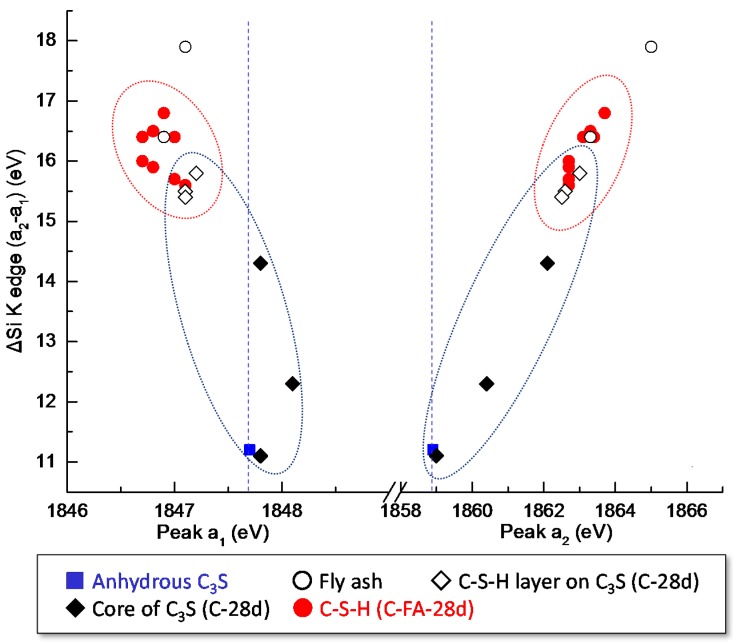
Relationship between the peak positions (a_1_ and a_2_) and the energy separation values (a_2_ − a_1_) in the selected areas of C-28 and C-FA-28d. The energy resolution of the spectrum is ±0.1 eV. Peak positions and separation values used for plotting are tabulated in Table 2.

All peak positions (a_1_ and a_2_) and energy separations (a_2_ − a_1_) of the selected areas of C-28 and C-FA-28d are shown in Table 3 and are plotted in Figure 10. After 28 days of hydration, the C–S–H layer in the pure C_3_S system had a lower a_1_ peak energy and higher a_2_ peak energies than anhydrous C_3_S. Alternatively, the C–S–H formed in C_3_S-HVFA system had a much lower a_1_ peak energy and higher a_2_ peak energies than the C–S–H layer on C_3_S in the pure C_3_S system. A previous study [21] illustrated that the peak positions (both a_1_ and a_2_) that were shifted in the Si K edge of the synthetic C–S–H increased with a decrease in the Ca/Si. Considering the silicate polymerization of C–S–H, C–S–H in the HVFA system should exhibit a greater peak a_1_ value than the C–S–H layer in pure C_3_S system. Therefore, the lower a_1_ peak energy of C–S–H in the C_3_S-HVFA system is a direct indication of Al incorporation into C–S–H, which decreases the Si-O bond and the effective charge on the Si atoms in C–S–H, resulting in a Si 1s binding energy in the Si K edge NEXAFS spectrum [21]. Additionally, the higher a_2_ peak energies could be attributed to the enhanced degree of silicate polymerization in C–S–H due to HVFA incorporation.

### 3.4. ^27^Al MAS NMR Analysis

The ^27^Al MAS NMR technique has been used to differentiate between tetrahedral (Al^[IV]^) and octahedral (Al^[VI]^) coordination because of their different ^27^Al isotropic chemical shifts; the Al^[IV]^ signal occurs in the range of 50–100 ppm, and the Al^[VI]^ signal is observed in the 10–20 ppm range [18,22,44,45]. The ^27^Al NMR spectrum tends to be characterized by broad peaks due to the quadrupolar nucleus of ^27^Al, but the quadrupolar coupling parameters can help identify structurally distinct Al sites [46]. Skibsted et al. [47] further developed methods for monitoring the hydration of aluminate cements and used ^27^Al NMR spectroscopy to study the calcium aluminate phases in cements. The broad peaks often overlapped, but the Al^[VI]^ peak positions in AFt [37,44] and AFm [46] could be resolved and were proven to be approximately 13 ppm and 8 ppm, respectively, in hydrated pastes. A peak at approximately 66 ppm was assigned to Al^[IV]^ in C–S–H. 

**Figure 11:**
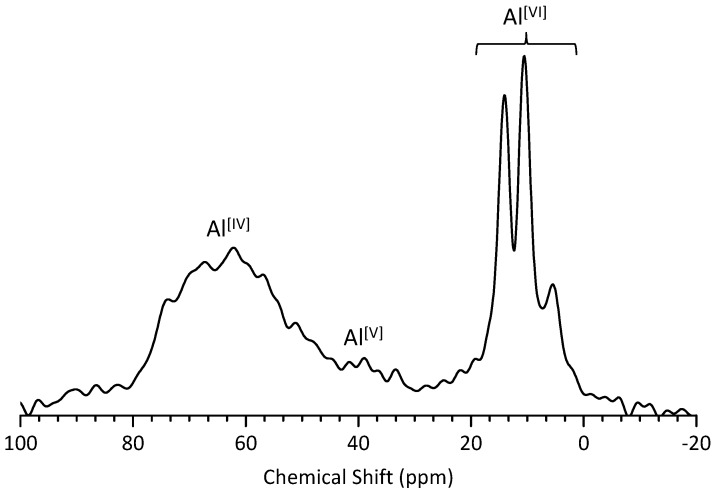
^27^Al MAS NMR spectroscopy of C-FA-28d.

As shown in Figure 11, the ^27^Al NMR spectra of C-FA-28d confirmed the presence of Al^[IV]^, Al^[V]^, and Al^[VI]^. Several sharp octahedral Al environments were observed in the ^27^Al NMR spectra of C-FA-28d. Specifically, three peaks were observed between 0 and 20 ppm: the AFt phase at ~14 ppm, AFm at ~10.5 ppm and a third aluminate hydrate (TAH) phase at ~5.6 ppm. A peak at approximately 40 ppm was also observed and was attributed to Al^[V]^. This Al species has been assigned to water-activated samples containing fly ash [48]. The broad resonance in the range of 50 to 80 ppm observed in this study is typical of Al^[IV]^ sites in less crystalline structures [15,22]. This Al^[IV]^ signal included Al in the anhydrous material and in C–S–H [18], where it was substituted for Si ions at the bridging tetrahedral sites. The Al^[IV]^ signal in synthetic C–S–H with a Ca/Si ratio of 1.0 occurs at 62.6 ppm [13], in agreement with the peak position (≈63 ppm) observed herein. Al^[IV^**^]^** in C–S–H decreases Ca/Si and results in the enhanced silicate polymerization and the increased silicate chain length of C–S–H [18,23,25]. This will be further discussed with ^29^Si MAS NMR and TEM-EDX analysis. 

### 3.5. ^29^Si MAS NMR Analysis

**Figure 12:**
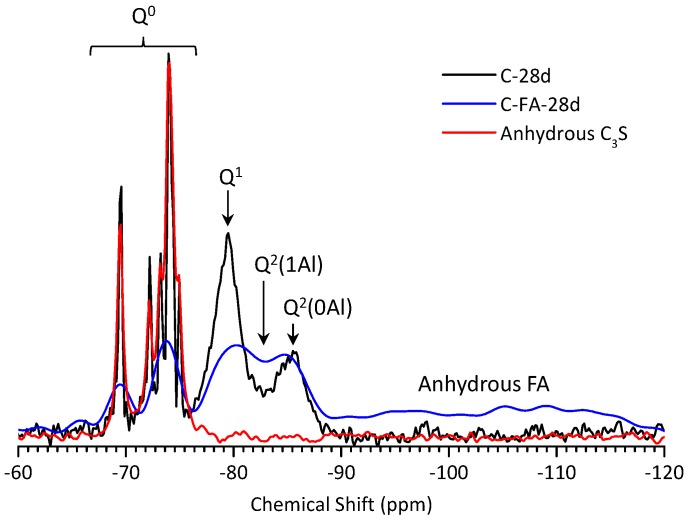
^29^Si MAS NMR spectroscopy of anhydrous C_3_S, C-28d, and C-FA-28d.

Figure 12 shows the ^29^Si MAS NMR spectra for anhydrous C_3_S (red line) and for the C-28d (blue line), and C-FA-28d (black line) pastes. The NMR spectrum of anhydrous C_3_S has a number of resonances in the range of from −68 to −76 ppm all with chemical shifts typical of an orthosilicate (Q^0^) [49,50]. The multiple resonances were due to several isolated silicate anion environments [50]. ^29^Si MAS NMR studies have shown that C–S–H produced during PC hydration contains only chain-end (Q^1^) and middle-chain (Q^2^) units [12,51,52]. In agreement with the studies [49,53], the C-28d spectrum had peaks in the range of from −76 to −90 ppm, which were due to the Q^1^ and Q^2^(0Al) sites in C–S–H that appeared after C_3_S hydration.

Comparing the C-FA-28d blend with hydrated C-28d, the peak intensity of Q^0^ significantly decreased as a result of the incorporation of HVFA. Additionally, the NMR spectrum of C-FA-28d had much broader overlapping due to the presence of a large amount of paramagnetic Fe^3+^ ion provided from HVFA [12,54]. In addition to Q^1^ and Q^2^(0Al), a third hydrate peak was observed at approximately −82 ppm and was attributed to Q^2^(1Al) as previously reported [18,55]. Here, no Q^1^(1Al), which was expected to be discovered when Al substituted for Si at non-bridging sites, was found. This confirms that Al is substituted for Si only in the bridging tetrahedral units of C–S–H, as observed in the previous reports [13,22,26]. The C-FA-28d spectrum also had a broad peak at −109 ppm due to the cross-linking sites in the three-dimensional framework (Q^4^) of the anhydrous fly ash [12,52], showing that the fly ash did not fully react within 28 days. A peak was also observed at approximately −95 ppm in the C-FA-28d spectrum, which indicated the presence of chain branching sites (Q^3^). This resulted from either a continuous range of Q^n^(mAl) species in the fly ash glassy phase [56] or carbonation of the C–S–H phase. Because the thermogravimetric analysis results showed some carbonation of the C-FA-28d paste, the –95 ppm peak was attributed to Q^3^ and not Q^4^ with Al substitution, which could lead to a downfield peak shift.

**Figure 13:**
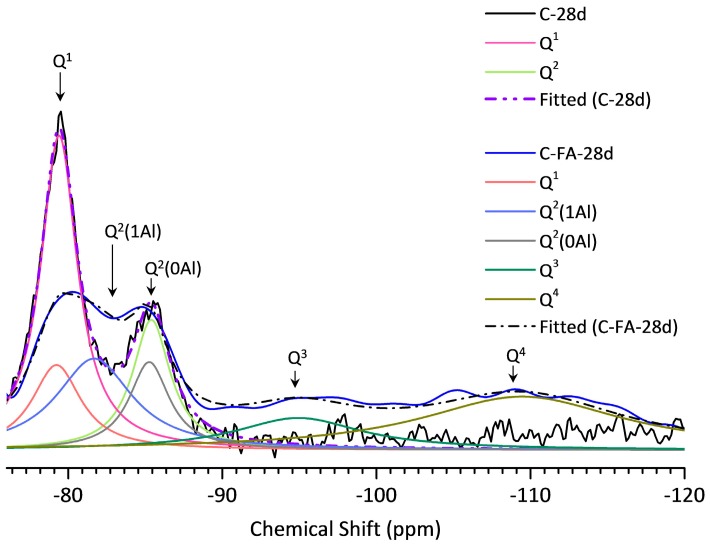
Deconvolution of the ^29^Si MAS NMR spectrum of C-28d and C-FA-28d.

**Figure 14:**
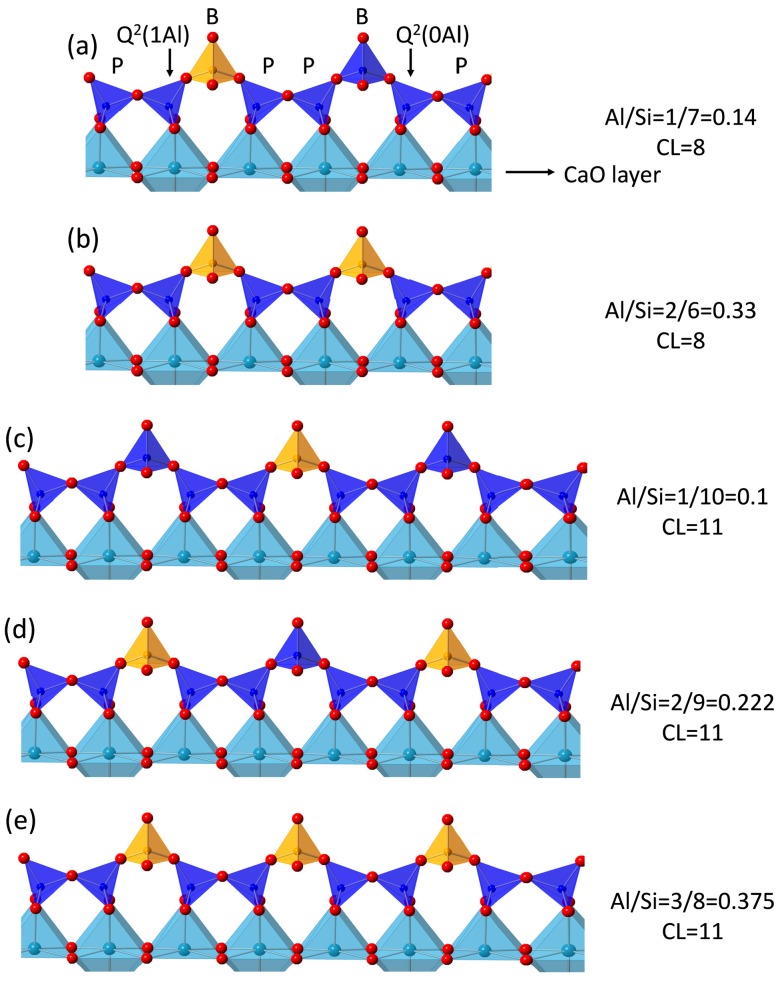
Schematics of the chain structure of Al substituted C–S–H with different chain lengths and Al/Si in the HVFA system. The silicate units are displayed as dark blue tetrahedrons, whereas the calcium and oxygen atoms are illustrated as light blue and red spheres, respectively.

The deconvoluted ^29^Si MAS NMR spectra of the hydrate phases of C-28d and C-FA-28d obtained from the fitting are presented in Figure 13. Based on the relative peak intensities obtained by deconvoluting the spectra, the silicate MCLs and Al/Si ratios of C-28d and C-FA-28d were calculated (Table 4). C-28d had an MCL of 2.53, which is consistent with the MCL of 2.4–2.6 for 50% to 70% hydrated C_3_S [57], whereas C-FA-28d had an MCL that was 4 times longer (10.26) than that of C-28d, demonstrating that the degree of silicate polymerization was higher in the HVFA system. The longer silicate chain length of C–S–H in C-FA-28d could also be due to the increased Al^[IV]^ occupancy in C–S–H, as reported previously [18,23,25]. Schematic representations of the C–S–H chain structures with different chain lengths (CLs) and Al/Si ratios in the HVFA system are illustrated in Figure 14. The schematic C–S–H structure in the HVFA system is based on the atomic structure of tobermorite with the following assumptions. First, C–S–H in the HVFA system has a single silicate chain, and Al substitutes for Si only at the bridging (B) tetrahedral sites and not at the paired (P) or end-chain tetrahedral sites. Second, the C–S–H consists of both dimeric and higher polymeric species, mainly linear pentamers (CL = 5) and octamers (CL = 8), suggesting a 2, 5, 8… (3n − 1) sequence of linear silicate chain lengths [12,58]. Third, no Q^3^ sites that link two dreierketten chains together exist in the C–S–H. Based on the calculated MCL (10.26), Al/Si ratio (0.257), relative ratios between the Q^n^ species (Q^1^:Q^2^(0Al):Q^2^(1Al)) measured by ^29^Si NMR, and the assumptions described above, Al is likely to substitute for Si in at least two of the three bridging tetrahedral sites in the C–S–H silicate chain (Figure 14d). Both ^27^Al and ^29^Si NMR analysis confirmed that incorporating HVFA into the C_3_S system induces the increased uptake of Al (Al^[IV]^) in C–S–H which results in an enhanced silicate chain length in C–S–H.

### 3.6. Morphological TEM Imaging of the C_3_S-HVFA Paste

**Figure 15:**
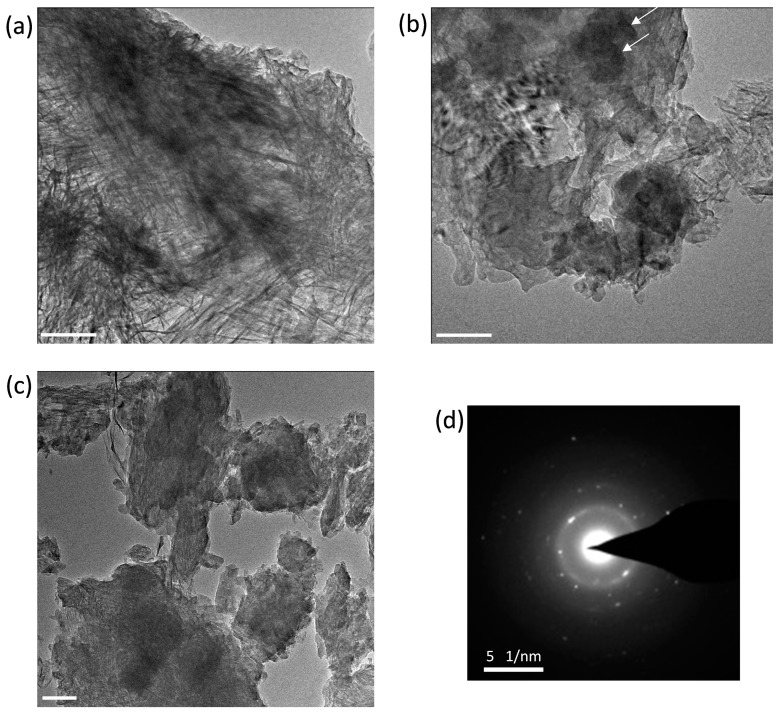
TEM image of C–S–H in C-FA-28d paste. (**a**) Fine OP C–S–H; (**b**,**c**) coarse C–S–H; and (**d**) SAED image pattern of Ca(OH)_2_ in (**c**). The scale bars in (**a**–**c**) represent 100 nm. The large Ca(OH)_2_ crystals are indicated by the arrows in (**b**). The SAED image of the sample presents a crystalline phase assumed to be nanocrystalline Ca(OH)_2_ intermixed with the C–S–H phase.

TEM images of C–S–H in the hydrated C_3_S particles in C-FA-28d are shown in Figure 15. The images show the fully reacted particles and the fine and coarse C–S–H in the hydrated C_3_S particles. As expected from the STXM and NMR results, no anhydrous C_3_S was observed in the images. Furthermore, fine C–S–H (Figure 15a) and large Ca(OH)_2_ crystals intermixed with the C–S–H (Figure 15b) were observed. The presence of intermixed Ca(OH)_2_ nanocrystals and C–S–H in Figure 15c was confirmed by SAED, as shown in Figure 15d. The EDX chemical analysis performed on the sample resulted in an approximate calculated Ca/Si ratio of 1.13, which was much less than that of the C–S–H layer on the pure hydrated C_3_S particles, which ranged from 1.5 to 1.9 [12,59,60]. The decreased Ca/Si ratio of C–S–H in the HVFA system indicated that the increased silicate polymerization of C–S–H increased in the HVFA system, which is in good agreement with the STXM and NMR results. 

## 4. Conclusions

This study investigated the effects of HVFA incorporation on the C_3_S hydration kinetics, as well as the morphology and atomic binding structure of C–S–H. The formation of Ca(OH)_2_ in the C_3_S-HVFA paste was significantly delayed during the early stages of C_3_S hydration. HVFA lengthens the induction period of the C_3_S hydration process at the early age; however, the degree of C_3_S hydration was enhanced by the use of HVFA after 3 days of hydration. The increased degree of hydration was presumably due to the enhanced C_3_S dissolution and precipitation of hydration products upon HVFA incorporation. 

Direct evidence of Al incorporation into C–S–H in the C_3_S-HVFA system was provided by the ^27^Al MAS NMR, which showed that Al substituted for Si only at the C–S–H bridging tetrahedral sites in the C_3_S-HVFA system. Additionally, ^29^Si MAS NMR showed that in the C_3_S-HVFA system, C–S–H had a silicate chain length that was 4 times longer than that in the pure C_3_S system (10.26 vs. 2.53), which confirms the higher degree of silicate polymerization in the C_3_S-HVFA system. The Al/Si (0.257) and the calculated silicate chain length suggested that tetrahedrally coordinated Al is likely to be incorporated in at least two of the three bridging tetrahedral sites in the C–S–H silicate chains produced in the C_3_S-HVFA system. The smaller Ca/Si (1.13) estimated from the TEM-EDX data also demonstrated the increased Al incorporation and degree of silicate polymerization of C–S–H in the C_3_S-HVFA system.

For the first time STXM was used to characterize the C–S–H in the C_3_S and C_3_S-HVFA systems. STXM imaging showed that the Si concentration of the C–S–H in the C_3_S-HVFA system was very uniform whereas apparent C–S–H layers were observed on only the C_3_S particles in the pure C_3_S system. While the formation of the C–S–H layer on C_3_S in the pure C_3_S system hindered the hydration of C_3_S resulting in an anhydrous core area, most of the C_3_S particles in the C_3_S-HVFA system were fully hydrated, and no obvious C–S–H layers were observed. This was due to the increased C_3_S dissolution in the C_3_S-HVFA system during the early stages of hydration, as observed in the thermal analyses. As a result of the enhanced hydration, the C–S–H in the C_3_S-HVFA system had a lower a_1_ peak energy and higher a_2_ peak energies at the Si K edge in the NEXAFS spectra than the C–S–H layers in the neat C_3_S system. The lower a_1_ peak energy of C–S–H in the HVFA system was conclusive evidence of Al substitution for Si in C–S–H, and the higher a_2_ peak energies were indicative of the enhanced degree of silicate polymerization due to the additional silicate provided by HVFA. In contrast, the Al incorporation and the enhanced silicate polymerization did not significantly influence the Ca L_III,II_ edge NEXAFS spectra of the CaO layer of C–S–H in the HVFA system. The results measured by STXM are in good agreement with the previous findings determined by other characterization methods. The findings above lead to the conclusion that, in order to enhance the understanding of C_3_S-HVFA system, it is necessary to further investigate the effect of fly ash containing an abundance of CaO on the morphology, the atomic binding structure of C–S–H and the kinetics of C_3_S hydration.

## Figures and Tables

**Table 1:** Oxide composition of Class F fly ash.

Oxide Compositions (wt %)	SiO_2_	Al_2_O_3_	Fe_2_O_3_	CaO	MgO	Na_2_O	K_2_O	P_2_O_5_	TiO_2_	MnO	Loss on Ignition
Class F Fly Ash	62.00	18.90	4.90	5.98	1.99	2.41	1.14	0.26	1.09	0.04	1.30

**Table 2:** Ca L_III,II_ edge peak positions and splitting energy of anhydrous C_3_S, Ca(OH)_2_, and regions of interest (ROIs) in C-28d and C-FA-28d.

Sample	Area	Peak Positions (eV)					Splitting of L_III_ and L_II_ (eV)	
		a_1_	a_2_	a_3_	b_1_	b_2_	∆Ca-L_III_ (a_3_ − a_2_)	∆Ca-L_II_ (b_2_ − b_1_)
Anhydrous C_3_S	-	346.7	347.7	349.0	351.1	352.3	1.3	1.2
Ca(OH)_2_	-	346.6	347.6	349.1	351	352.4	1.5	1.4
C-28d	Area 1	346.9	348.0	349.1	351.4	352.4	1.1	1
C-FA-28d	Area 2	346.8	348.0	349.1	351.3	352.4	1.1	1.1
	Area 3	346.9	348.0	349.1	351.3	352.4	1.1	1.1
	Area 4	346.8	348.0	349.1	351.3	352.4	1.1	1.1

**Table 3:** Si K edge peak positions and energy separation values.

Sample	Area	Peak a_1_ (eV)	Peak a_2_ (eV)	ΔSi-K (a_2_ − a_1_) (eV)
Anhydrous C_3_S	-	1847.7	1858.9	11.2
C-28d	Area 1	1848.1	1860.4	12.3
	Area 2	1847.2	1863.0	15.8
	Area 3	1847.8	1859, 1862.1	11.1, 14.3
	Area 4	1847.1	1862.6	15.5
	Area 5	1847.1	1862.5	15.4
C-FA-28d-1	FA	1847.1	1865	17.9
	Area 1	1846.9	1863.7	16.8
	Area 2	1847.1	1862.7	15.6
	Area 3	1847	1862.7	15.7
	Area 4	1846.8	1863.3	16.5
C-FA-28d-2	FA	1846.9	1863.3	16.4
	Area 1	1847.0	1863.4	16.4
	Area 2	1846.8	1862.7	15.9
	Area 3	1846.7	1862.7	16
	Area 4	1846.7	1863.1	16.4

**Table 4:** Deconvolution results of C-28d and C-FA-28d.

Sample	Peak	Shift	Intensity (%)	MCL	%B	Al/Si
C-28d	Q^1^	−79.51	78.9	2.53	-	-
	Q^2^(1Al)	-	-			
	Q^2^	−85.51	21.1			
C-FA-28d	Q^1^	−79.30	12.95	10.26	76.3	0.257
	Q^2^(1Al)	−81.77	27.20			
	Q^2^	−85.35	12.69			
	Q^3^	−94.93	15.88			
	Q^4^	−109.45	31.29			
